# Reliability as a Precondition for Trust—Segmentation Reliability Analysis of Radiomic Features Improves Survival Prediction

**DOI:** 10.3390/diagnostics12020247

**Published:** 2022-01-19

**Authors:** Gustav Müller-Franzes, Sven Nebelung, Justus Schock, Christoph Haarburger, Firas Khader, Federico Pedersoli, Maximilian Schulze-Hagen, Christiane Kuhl, Daniel Truhn

**Affiliations:** 1 Department of Diagnostic and Interventional Radiology, University Hospital Aachen, 52074 Aachen, Germany; gustav.mueller-franzes@rwth-aachen.de (G.M.-F.); sven.nebelung@med.uni-duesseldorf.de (S.N.); firas.khader@rwth-aachen.de (F.K.); fpedersoli@ukaachen.de (F.P.); mschulze@ukaachen.de (M.S.-H.); ckuhl@ukaachen.de (C.K.); dtruhn@ukaachen.de (D.T.); 2 Department of Diagnostic and Interventional Radiology, University Hospital Düsseldorf, 40225 Düsseldorf, Germany; 3 Department of Research and Development, CheckupPoint GmbH, 81669 Munich, Germany; ch@checkuppoint.com

**Keywords:** radiomic features, overall survival, segmentation variability, inter-rater reliability, neural network, robustness

## Abstract

Machine learning results based on radiomic analysis are often not transferrable. A potential reason for this is the variability of radiomic features due to varying human made segmentations. Therefore, the aim of this study was to provide comprehensive inter-reader reliability analysis of radiomic features in five clinical image datasets and to assess the association of inter-reader reliability and survival prediction. In this study, we analyzed 4598 tumor segmentations in both computed tomography and magnetic resonance imaging data. We used a neural network to generate 100 additional segmentation outlines for each tumor and performed a reliability analysis of radiomic features. To prove clinical utility, we predicted patient survival based on all features and on the most reliable features. Survival prediction models for both computed tomography and magnetic resonance imaging datasets demonstrated less statistical spread and superior survival prediction when based on the most reliable features. Mean concordance indices were C_mean_ = 0.58 [most reliable] vs. C_mean_ = 0.56 [all] (*p* < 0.001, CT) and C_mean_ = 0.58 vs. C_mean_ = 0.57 (*p* = 0.23, MRI). Thus, preceding reliability analyses and selection of the most reliable radiomic features improves the underlying model’s ability to predict patient survival across clinical imaging modalities and tumor entities.

## 1. Introduction

The application of machine-learning methods in clinical radiology has gained significant momentum in recent years. One such method that is often used is radiomic analysis.

In the early days of radiomic analyses, hopes were high that the all-embracing approach of casting as much information as possible into exploitable numeric quantities would lead to radiomic breakthroughs in disease prognostication, treatment prediction and tumor grading [1]. However, until now, applications of radiomics in clinical radiology have fallen short of expectations and results are often neither reproducible nor transferrable. The reasons are manifold [2]: First, image acquisition and reconstruction parameters, such as tube currents for CT or reconstruction algorithms, differ between sites, which brings about variable image characteristics and renders inter-site transferability difficult, let alone inter-vendor and inter-platform transferability. Second, software frameworks for the calculation of radiomic features may calculate those features differently depending on their implementation. These aspects of lacking standardization have received increasing attention recently [3] and are now being addressed by the image biomarker standardization initiative [4]. Third, radiomic analyses are always performed on parts of the image—in most cases tumors—that first have to be outlined by an expert. Correct segmentation of tumor boundaries is a critical step for subsequent analyses, because inter-reader variability is inevitable and radiomic signatures are affected by variations in segmentation outlines [5]. Evaluating the interdependence of segmentation precision and radiomic features is labor intensive and has thus only been studied on small datasets [6,7]. Consequently, comprehensive analyses of radiomic variability brought about by variable segmentations have thus far been missing.

In this work, we aim to close this gap by (i) providing a full-scale analysis of radiomic feature reliability in five clinical image datasets of clinical tumor entities across the pertinent cross-sectional imaging modalities, CT and MRI, and by (ii) systematically assessing the interrelatedness of radiomic feature reliability and the underlying models’ ability to predict patient survival based on imaging.

To this end, we hypothesized that (i) the reliability of a large variety of radiomic features may be assessed across different clinical imaging modalities and tumor entities and that (ii) preceding reliability analyses and deliberate selection of radiomic features render machine-learning algorithms more accurate and reliable by improving their predictive capabilities.

Our work demonstrates that radiomic features are sensitive to segmentation variability and that a pre-selection of robust radiomic features might improve the ability of machine learning models to predict survival of patients.

## 2. Materials and Methods

### 2.1. Experimental Design

We conducted this study in three phases. First, we trained a dedicated neural network to introduce variability in the segmentation outlines by learning and systematically varying how experts would delineate tumor outlines [8]. Thereby, sets of variable yet realistic segmentation outlines for each tumor in diverse clinical image datasets were generated. Second, radiomic features for each segmentation were calculated. Third, we analyzed the stability of radiomic features on the datasets using the intra-class correlation coefficient (ICC) and observed the effect of preceding reliability analyses and deliberate radiomic feature selection on the survival predictions based on the CT and MRI datasets’ clinical tumor entities, using machine learning. To this end, only those datasets were included for which additional survival data were available, i.e., the CT datasets of the 421 patients suffering from non-small cell lung cancer and the MRI datasets of the 335 patients suffering from brain tumors. Approval to perform machine learning analyses on the available pseudonymized data were granted by the local Ethical Committee.

### 2.2. Data

Five datasets, for which the respective details and references are given in Table 1, were used in this prospective exploratory study. These datasets comprised a total of 3158 manual tumor segmentation outlines (n = 2823 [CT datasets]; n = 335 [MRI datasets]) of 1972 patients (n = 1637 [CT datasets]; n = 335 [MRI datasets]): (i) the Non-Small-Cell-Lung-Cancer (NSCLC) dataset containing 487 outlines of non-small cell lung cancer in the CT scans of 421 patients; (ii) the Lung Image Database Consortium image collection (LIDC) dataset containing 1175 outlines of lung cancer in the CT scans of 875 patients; (iii) the Liver Tumor Segmentation (LiTS) dataset containing 908 outlines of hepatic tumors in the CT scans of 131 patients; (iv) the Kidney Tumor Segmentation (KiTS) dataset containing 253 outlines of kidney tumors in the CT scans of 210 patients; and (v) the Brain Tumor Segmentation (BraTS) dataset containing 335 outlines of brain tumors in the MRI scans of 335 patients. For our analysis, we used the fluid-attenuated inversion recovery (FLAIR) sequence. Please note that the underlying acquisition protocol of the MR images is different for each participating institution and representative of actual clinical protocols [9].

**Figure 1:**
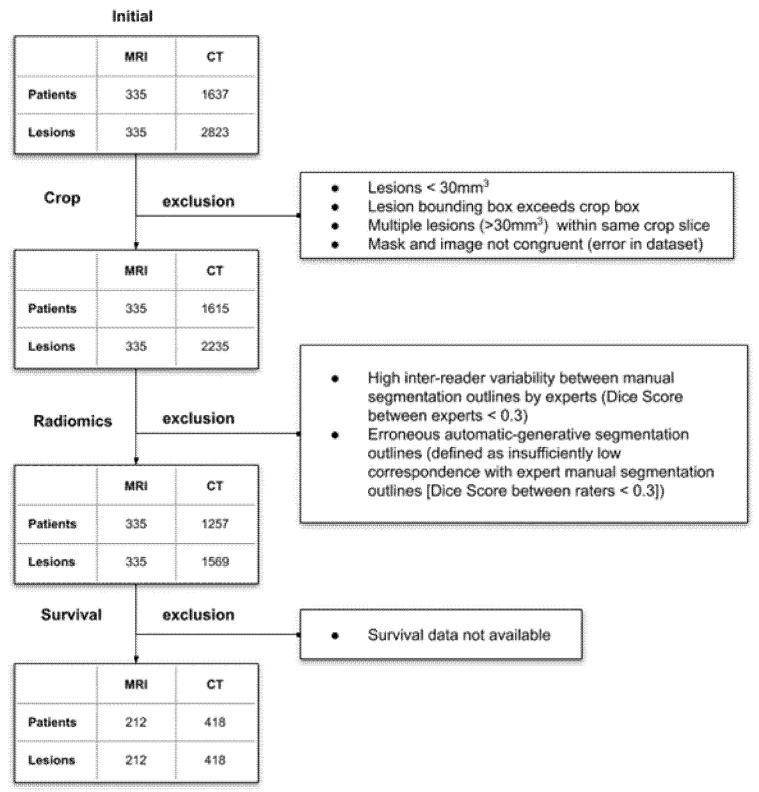
Flow chart showing patient and lesion details as well as exclusion criteria for each step of the post-processing pipeline. NN—Neural Network. A more detailed visualization of the data processing pipeline can be found in Appendix A Figure A1.

All of these datasets were used in the first two study phases, while datasets (i) and (v) were used for prediction of survival because additional survival data were only available for these datasets. Figure 1 details the data flowchart as well as relevant exclusion criteria. All of the datasets have been previously published [10,12,14,15,17], however a comprehensive reliability analysis of the complete datasets has not yet been performed.

### 2.3. Training of the Segmentation Network

Each dataset contained manually delineated expert tumor outlines. To obtain additional variable tumor outlines, we used a recently validated neural network designed to capture and reflect uncertainty in medical image segmentation using probabilistic hierarchical segmentation (PHiSeg) [8]. Hyperparameters were selected as in earlier studies [18]. Practically, images with tumor lesions were cropped before training using standard routines in python. To this end, a rectangular bounding box was placed around the lesions with the bounding box centered on the lesion. Care was taken to ensure that the lesion outlines were completely contained in the bounding box. Bounding box sizes were variable and determined beforehand by systematic analysis of tumor size distributions in the respective dataset. More specifically, bounding boxes were dimensioned to be larger than the mean plus two times the standard deviation of the lesions’ maximum diameters. Consequently, bounding boxes of 128 × 128 pixels (datasets (i) and (ii)) and 192 × 192 pixels (datasets (iii)–(v)) were generated. Outlier lesions that were too small (i.e., <30 mm^3^) or too large (i.e., exceeding bounding boxes), and multiple lesions within the same crop slice were excluded from further analysis. The pre-trained neural network was subsequently used to automatically generate 100 additional segmentation outlines. To this end, the segmentation outlines were varied using a hierarchical probabilistic model. The resulting segmentations are referred to as automatic-generative segmentation outlines from here.

### 2.4. Plausibility of Segmentation Outlines

To assess if the automatic-generative segmentation outlines are representative of the radiomic features’ natural variability, we performed the following evaluations:

First, plausibility of the automatic-generative segmentation outlines was checked visually by two experienced clinical radiologists (SN and DT with 5 and 8 years of experience each) by going through a set of 40 representative lesions in each dataset.

Second, radiomic feature variability was compared between manual and automatic-generative segmentation outlines [8] for dataset (ii), i.e., the LIDC dataset. For this dataset, four manual expert segmentation outlines were available for each lesion. The ICC was calculated for all radiomic features within the group of manual and automatic-generative segmentation outlines, respectively, by use of the open-source SciPy library [19] implemented in Python (v3.7.4, Python Software Foundation). Of note, we employed the ICC (1) as defined and recommended by Liljequist et al. [20]. Then, radiomic features were grouped into four categories according to their ICCs, i.e., highly unstable (25% of least consistent features [quartile 4]), moderately unstable (25% to 50% of least consistent features [quartile 3]), moderately stable (25% to 50% of most consistent features [quartile 2]), and highly stable (25% of most consistent features [quartile 1]). Correspondingly, ICCs were calculated and grouped for the automatic-generative segmentation outlines, too.

### 2.5. Feature Computation

For each tumor segmentation outline, radiomic features were extracted on the 2D axial image containing the greatest tumor cross-sectional area. We employed the PyRadiomics toolkit implemented in Python [21] to extract radiomic features from the following feature groups: First-Order, Shape, Gray Level Co-occurrence Matrix, Gray Level Size Zone, Gray Level Run Length Matrix and Gray Level Dependence Matrix including wavelet features. In total, 439 features were extracted for each lesion. Please refer to https://pyradiomics.readthedocs.io/en/latest/ (accessed on 5 December 2021) for in-depth documentation on these radiomics feature groups and individual features. In short, first order parameters describe the histogram of voxel intensities using common metrics such as mean, median, variance, uniformity, skewness and kurtosis. The gray level matrices describe the relationship between intensities and their spatial distribution.

### 2.6. Feature Selection

Radiomic feature reliability was assessed based on the ICC. Radiomic features were ranked according to their ICC values based on the 100 automatic-generative segmentation outlines (all datasets) as well as the manual segmentation outlines (dataset (ii)/LIDC). Since the ICC calculation is based on multiple segmentation outlines for the same lesion, a comparison between automatic-generated and manual segmentation outlines was only made for the LIDC dataset because only this dataset had multiple expert segmentation outlines (four per lesion) available. Features were then grouped into three categories [20,22,23]:

“high ICC”, containing all features with an ICC value >0.99;
“low ICC”, containing all features with an ICC value <0.75;
“all ICC”, containing all features irrespective of the ICC values.

Of note, we chose a relatively high threshold of 0.99 for the high-ICC group to study the effects of very stable radiomic features when predicting clinical outcomes. Thereby, the numbers of features in the high- and low-ICC groups were secondarily balanced. Based on these sets, three distinct survival models were trained as defined below. Within each of these models, features were further selected with sequential forward selection. This means that the first radiomic feature was selected as having the highest univariate concordance index (C-index), i.e., the highest power in predicting survival. Closely related to the area under the ROC curve, the C-index is a global estimate of the respective model’s discriminatory power, i.e., its ability to predict survival times based on the radiomic features. C-indices of 1.0 and 0.5 indicate perfect and random model predictions, respectively.

### 2.7. Survival Prediction

To predict survival based on the radiomic features, the proportional hazard model according to Cox [24] was used. The performance of the models was evaluated based on the C-indices and the radiomic features extracted from the manual expert segmentation outlines (n = 1) and the additional automatic-generative segmentation outlines (n = 100).

Patients available for datasets (i) (NSCLC dataset, CT) and (v) (BraTS dataset, MRI) were partitioned into high- and low-risk groups. Thus, each patient underwent individual image-based risk score quantification and was either allocated to the low-risk group (if the risk score was below the median) or the high-risk group (if above). Survival data were evaluated separately for those two groups.

### 2.8. Statistical Analysis

Python and its scikit-survival package [25], which is a library for time-to-event analyses, were used and data were split into training- and test-sets in five-fold stratified cross-validation, i.e., 80% and 20%, respectively. Survival times were stratified in one-year intervals, i.e., ≤1 year, 1–2 years, 2–3 years, and ≥3 years. Further stratification was introduced by determining whether the event, i.e., death, was observed or censored. In a comprehensively commented format, the code is made publicly available in a GitHub repository under https://github.com/mueller-franzes/ReliableRadiomics (accessed on 5 December 2021).

Friedman’s test was used as an a-priori test to assess the differences between the C-indices of the three groups (high ICC, all features and low ICC). To assess differences between pairs of groups, Wilcoxon’s signed rank test was used. As a side note, we employed Wilcoxon’s test instead of a paired t-test as D’Agostino’s test for normality failed.

Bartlett’s test was used to compare the variance in C-indices. Due to this study’s exploratory design and because for each modality, two comparisons were performed, the Bonferroni-corrected level of significance was set to 0.05/2.

## 3. Results

### 3.1. Lesion Segmentation by the Neural Network

**Figure 2:**
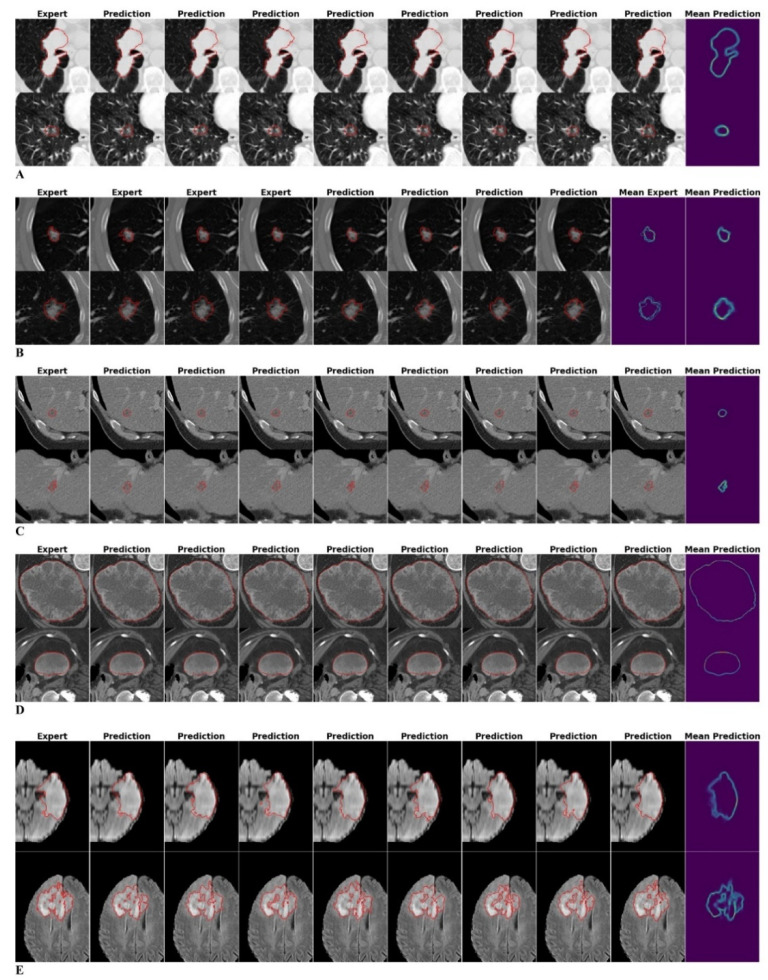
Manual expert and automatic-generative neural network-based segmentation outlines of representative lesions of the NSCLC (Non-Small Cell Lung Cancer) (**A**), LIDC (Lung Image Database Consortium) (**B**), LITS (Liver Tumor Segmentation) (**C**), KITS (Kidney Tumor Segmentation) (**D**), and BRATS (Brain Tumor Segmentation) (**E**) datasets. The rightmost column shows an overlay of all available segmentation outlines. Only the LIDC dataset provides four different manual segmentation outlines (by experts) per lesion whose overlay is shown as the additional column titled “Mean Expert”. Overall, neural network-based predictions produce realistic and robust segmentation outlines. Variability in automatic-generative segmentation outlines is increased in tumor regions where delineation is inherently difficult.

Manual and automatic-generative segmentation outlines are given for representative tumor lesions of datasets (i) to (v) (Figure 2). Two experienced radiologists assessed whether the network’s delineation of tumor boundaries represents human-like segmentation performance. In their subjective evaluation of 40 tumor lesions of each dataset, it was found that in all areas where the human readers’ delineation of tumor boundaries would be challenging (e.g., due to fuzzy tumor boundaries), the neural network (NN)-based automatic-generative segmentation outlines demonstrated higher variability, while tumor delineation that was less challenging for experts (due to clear tumor boundaries against the background parenchyma) the NN based segmentation demonstrated consistent tumor boundaries.

**Figure 3:**
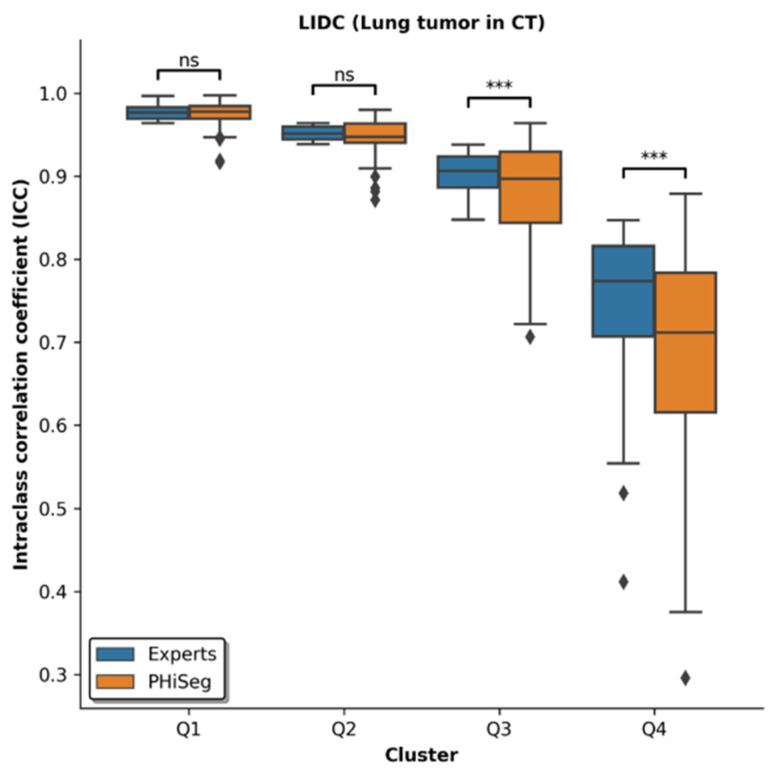
ICCs of radiomic features when calculated based on manual segmentations of lung lesions by four experts (blue) and 100 automatic-generative segmentation outlines generated by the pretrained neural network PHiSeg (orange). Features were grouped into quartiles based on manual expert segmentations and Q1 indicates the 25% most stable features, Q2 the 25–50% most stable features, Q3 the 25–50% least stable features, and Q4 the 25% least stable features. Overall, ICCs were largely similar, even though the NN introduced more variability. ns: not significant, ***: *p* < 0.001.

Overall, in this subjective evaluation, the NN was capable of representing the variability in radiomic features caused by differences in the segmentation outlines. For both manual and automatic-generative segmentation outlines in the LIDC dataset, ICCs were similar by trend, while the NN introduced statistically larger variability for the least stable features (Figure 3).

### 3.2. Reliability of Features

**Figure 4:**
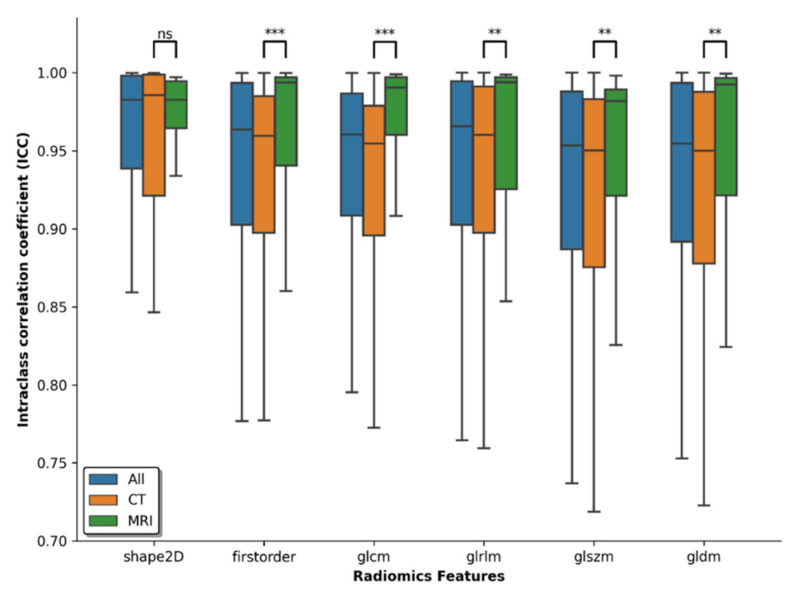
Reliability of radiomic features as a function of feature group and imaging modality. Features were grouped into shape features (“shape2D”), first-order features (“firstorder”), and gray-level matrix features, i.e., Gray Level Co-occurrence Matrix (“glcm”), Gray Level Run Length Matrix (“glrlm”), Gray Level Size Zone (“glszm”), and Gray Level Dependence Matrix (“gldm”). Imaging modalities are color-coded as follows: orange, only CT data; green, only MRI data; blue, all (CT and MRI). No consistent trends were found across the datasets, thus stressing the importance of analyzing feature reliability individually for each feature and modality. Asterisks denote statistical differences between the CT and the MRI dataset. ns: not significant, ** *p* < 0.01, *** *p* < 0.001.

Using ICCs to quantify reliability of features, we found that shape features were most reliable for radiomic features extracted from CTs with a median ICC of 0.986 [Q1 = 0.921, Q4 = 0.999]. First-order and gray-level-matrix features had median ICCs of 0.960 [Q1 = 0.897, Q4 = 0.985] and 0.953 [Q1 = 0.888, Q4 = 0.985], respectively. For MRI, first-order features were most stable with a median ICC of 0.994 [Q1 = 0.940, Q4 = 0.997]. Gray-level matrix and shape features had median ICCs of 0.988 [Q1 = 0.931, Q4 = 0.997] and 0.983 [Q1 = 0.964, Q4 = 0.995], respectively. Overall, no clear feature group- or imaging modality-related trends could be established for the feature classes (Figure 4).

Supplementary Table S1 gives a detailed account of the reliability of every single radiomic feature and every dataset. Please note that the data are also provided in machine-readable format (.csv) for use by fellow research groups to build upon our findings and include reliability analyses of radiomic features into their studies.

### 3.3. Survival Analysis Employing Feature Reliability

To test whether feature reliability analysis can help in building more consistent machine-learning models, we used radiomic feature analyses to predict survival in patients suffering from non-small cell lung cancer based on CT datasets (dataset (i)) and in patients suffering from brain tumors based on MRI datasets (dataset (v)).

If radiomic survival analysis was based on the high-ICC features (n = 77) only, the C-indices were more stable than when all radiomic features (n = 439) were taken into account. Significant differences in the C-index standard deviations (*C*_SD_) were found for the MRI dataset: dataset (v), *C*SD = 0.025 [high ICCs] vs. *C*SD = 0.030 [all ICCs], *p* < 0.001. For the CT dataset, these differences only tended towards significance: dataset (i), *C*SD = 0.024 [high ICCs] vs. *C*SD = 0.026 [all ICCs], *p* = 0.06.

In both datasets, the mean C-indices (*C*_mean_) were also higher if only high-ICC features were used to train the underlying models, yet significant differences were only found for the CT dataset: dataset (i), *C*_mean_ = 0.58 [high ICCs] vs. *C*_mean_ = 0.56 [all ICCs], *p* < 0.001; dataset (v), *C*_mean_ = 0.58 [high ICCs] vs. *C*_mean_ = 0.57 [all ICCs], *p* = 0.23.

**Figure 5:**
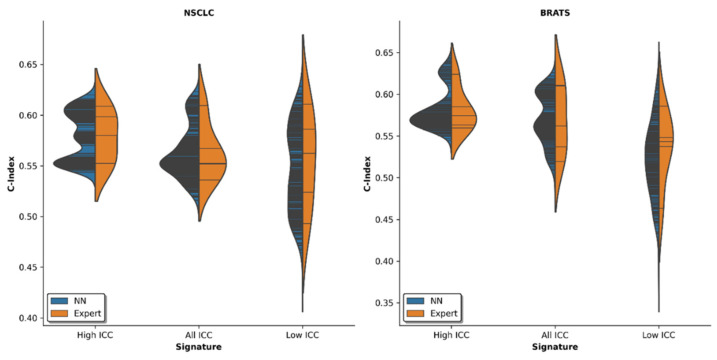
Violin plot of the Concordance indices (c-indices). Data are given as lines, while the filled curves give an estimate of the continuous probablitiy density. The violin plots indicate the predictive model’s discriminatory power as a function of ICC signature. Left: NSCLC (Non-Small Cell Lung Cancer, CT) dataset, right: BraTS (Brain Tumor Segmentation, MRI) dataset. C-indices were determined based on one manual expert and 100 automatic-generative segmentation outlines in five-fold cross-validation using a pretrained neural network (NN). The survival analysis based on only the most reliable radiomic features (as indicated by high ICCs) exhibited consistently higher mean C-indices (*p* < 0.001 for NSCLC and *p* = 0.23 for MRI) and lower variability (*p* = 0.06 for CT and *p* < 0.001 for MRI) as compared to all radiomic features or the least reliable radiomic features (*p* < 0.001 for NSCLC and *p* < 0.001 for MRI for the comparison of C-indices and *p* < 0.001 for NSCLC and *p* < 0.001 for MRI for the comparison of variability).

When the models were trained on only the low ICC features (n = 25), the C-indices were both significantly lower (dataset (i), *C*_mean_ = 0.54, *p* < 0.001; dataset (v): *C*_mean_ = 0.52, *p* < 0.001) and more spread out (dataset (i), *C*_SD_ = 0.042, *p* < 0.001; dataset (v), *C*_SD_ = 0.046, *p* < 0.001) when compared to all features. For both datasets, Figure 5 details the C-indices as a function of the ICC signature.

## 4. Discussion

This work focused on reliability analyses of radiomic features and their applicability for machine-learning algorithms using clinical CT and MRI datasets. We used a recently developed neural network architecture to generate 100 variable automatic-generative segmentation outlines for various tumor lesions in CT and MRI datasets to calculate radiomic features.

Automatic-generative segmentation outlines were visually compared with manual expert segmentation outlines by radiologists, and exemplary segmentation outlines are given in Figure 2. In agreement with previous research [8], we found that the automatic segmentation outlines are highly similar to the manual segmentation outlines. This is also reflected by the respective ICC scores (Figure 3). In particular, the most stable 50% of features revealed no significant differences between automatic and manual segmentations. However, an increased discrepancy was found for the less stable features. Most likely, due to the inherently increased susceptibility to inter-reader variation, less stable features are characterized by lower and more variable ICC scores. As machine learning models performed similarly nonetheless (Figure 5), this limitation was still considered acceptable. Beyond this, the variability in segmentation outlines might be better captured by one hundred automatic segmentation outlines than by four manual segmentation outlines per lesion (as the latter are inherently prone to sampling errors), thus leading to the observed wider spread of ICC scores in the automatic segmentation outlines. Additionally, four manual segmentation outlines (as generated by four human raters) may not be enough to accurately estimate the reliability of features which also carries the risk of overestimating the stability of (in fact) unstable features. Hence, automatic methods capable of generating larger numbers of segmentation outlines may be better suited to assess reliability of radiomic features, both in radiology and beyond.

Based upon this comprehensive multimodality database, we produced an extensive analysis of inter-reader reliability in terms of ICC for each radiomic feature. Reliability analysis indicated that radiomic features were most reliably assessed individually, rather than in association with a feature group. To our knowledge, this is the first study to provide such a large-scale analysis on various tumor entities across both clinical CT and MRI datasets. Other groups have worked on smaller datasets and reported comparable results on subsets of the data when using smaller numbers of segmentation outlines [18,26,27]. For the NSCLC dataset, Kadoya et al. found a C-index of 0.625 for a multivariate radiomic feature model [28]. In contrast, Fu et al. reported a C-Index of 0.67 when applying neural networks on the BRATS dataset [29], thereby indicating that deep learning-based approaches may achieve higher C-indices. This finding is plausible as such approaches achieve better results given enough data [30], yet disallow comparisons with radiomic analyses as performed in the present study.

To study whether reliability analyses as performed in this study can improve the clinical utility of machine-learning algorithms, we trained a machine-learning model to predict survival times based on the radiomic features for two datasets: a CT dataset comprising patients with non-small-cell lung cancer and an MRI dataset comprising patients with brain tumors. For both datasets, we demonstrated that using only the most reliable features (as selected based on the very highest and close-to-perfect ICCs) improves predictive power in terms of higher correspondence and less variability as indicated by higher mean C-indices and lower standard deviations. Consequently, preceding radiomic feature analysis and appropriate preselection renders such models more reliable. Fellow research groups [31,32,33] have found similar C-indices ranging from 0.58 to 0.62 when training machine-learning algorithms on the same CT datasets albeit with much greater variability.

Our work has limitations: First, our analysis is based on 2D segmentations due to limited hardware capacity. Dedicated graphics processing units are necessary to comprise full 3D volumes during training of the segmentation network. Implementing 3D analysis might be a potential future research direction once sufficient computing power becomes available. Second, even though we visually assessed the automatic-generative segmentation outlines, the agreement between these and the manual expert segmentation outlines could only be quantified for one of the datasets for which multiple manual expert segmentations were available. Visual assessment confirmed that the neural network-based automatic-generative segmentation outlines were considered realistic and largely reflective of the segmentation outlines generated by expert human readers. Future research should focus on the extension to three-dimensional segmentations—as increasingly used with the advent of greater processing power—and on the evaluation of our results on other modalities, e.g., positron emission tomography. To foster such research endeavors, we have appended our results—in particular the database containing the ICCs for each radiomic feature and for each of the employed datasets—in machine-readable format as Supplementary Material to this manuscript. Third, the radiomic expression profile not only depends on the segmentation outlines as examined in this study, but also on the underlying image acquisition and post-processing parameters that are, in turn, dependent on the manufacturer and device type. In addition, patient motion, resampling and discretization of voxel values and other factors are relevant, too, as previously reported by others [3,22,34,35,36,37]. These effects have only been studied in isolation so that future research should also examine their respective interplay. These effects have so far only been studied in isolation and future research should also examine their respective interplay. The question of whether radiomic feature stability varies between tumor types remains unanswered as yet. In this work, we have concentrated on radiomic feature stability with a focus on CT and MRI. ICC scores (as based on both manual and automatic segmentation outlines) may be prone to overestimation as they may be lower. For example, the BRATS dataset comprises both low-grade gliomas and glioblastomas and variance between different tumor lesions may be greater than among similar lesion types. However, this type of bias affects both manual and automatic segmentation outlines and future research should thus focus on ICCs when using strictly consistent tumor lesions and imaging modalities.

## 5. Conclusions

In conclusion, preceding reliability analyses and selection of the most reliable radiomic features improve the underlying model’s ability to predict patient survival across clinical imaging modalities and various tumor entities. Thereby, this study suggests a feasible and effective approach to further reduce the variability in reported capabilities of machine-learning algorithms and complements previous work aimed at improving image biomarker standardization [4].

## Figures and Tables

**Table 1:** Characteristics of image datasets used in this study. Data are given where available. NA—not available. UNK—Unknown. * Given as means ± standard deviation. ** M = Man, W = Woman.

Dataset	Tumor Entity	Patients [n]	Lesions [n]	Age * [Years]	Sex **	Cropping [Pixels]	Modality	Reference
NSCLC	Non-small cell lung carcinoma	421	487	68 ± 10	290M, 131W	128 × 128	CT	[1,10,11]
LIDC	Lung cancer	875	1175	NA	NA	128 × 128	CT	[11,12,13]
LiTS	Liver tumor	131	908	NA	NA	192 × 192	CT	[14]
KiTS	Kidney tumor	210	253	58 ± 14	123M, 87W	192 × 192	CT	[15]
BraTS	Glioblastoma and Lower Grade Glioma	335	335	61 ± 13 UNK: 19	NA	192 × 128	MRI	[9,16,17]

**Figure A1:**
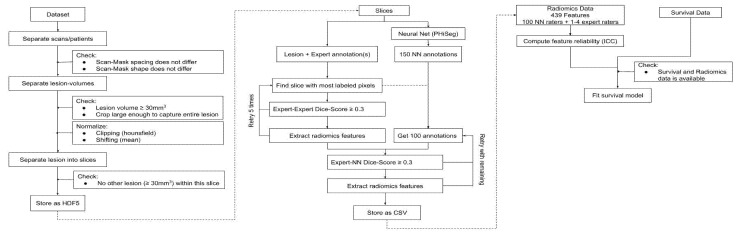
Data processing pipeline with inclusion and rejection criteria: first plausibility checks for the raw data were performed to ensure data integrity, then lesions were chosen that fulfill the inclusion criteria laid out in the text and slices were selected to be included in the study. Preprocessing for the BRATS data included normalizing of the z-scores (mean 0, standard deviation 1) and rescaling to [−500, 500] for the feature extraction. The Hounsfield units of the CT images were clipped as follows to: LITS and KITS [−200, 400], NSCLC [−1024, 200], LIDC [−500, 500].

## Data Availability

All datasets analyzed in the study are publicly available (Table 1). The source code of this manuscript is comprehensively commented and has been made publicly available on GitHub: https://github.com/mueller-franzes/ReliableRadiomics (accessed on 5 December 2021).

## Supplementary Materials

The following are available online at www.mdpi.com/article/10.3390/diagnostics12020247/s1, Table S1: ICC values of all 439 radiomic features for the five datasets used in our study.

## References

[1] Aerts H.J.W.L., Velazquez E.R., Leijenaar R.T.H., Parmar C., Grossmann P., Carvalho S., Bussink J., Monshouwer R., Haibe-Kains B., Rietveld D. (2014). Decoding Tumour Phenotype by Noninvasive Imaging Using a Quantitative Radiomics Approach. Nat. Commun..

[2] Kuhl C.K., Truhn D. (2020). The Long Route to Standardized Radiomics: Unraveling the Knot from the End. Radiology.

[3] Berenguer R., Pastor-Juan M.d.R., Canales-Vázquez J., Castro-García M., Villas M.V., Mansilla Legorburo F., Sabater S. (2018). Radiomics of CT Features May Be Nonreproducible and Redundant: Influence of CT Acquisition Parameters. Radiology.

[4] Zwanenburg A., Vallières M., Abdalah M.A., Aerts H.J.W.L., Andrearczyk V., Apte A., Ashrafinia S., Bakas S., Beukinga R.J., Boellaard R. (2020). The Image Biomarker Standardization Initiative: Standardized Quantitative Radiomics for High-Throughput Image-Based Phenotyping. Radiology.

[5] Rizzo S., Botta F., Raimondi S., Origgi D., Fanciullo C., Morganti A.G., Bellomi M. (2018). Radiomics: The Facts and the Challenges of Image Analysis. Eur. Radiol. Exp..

[6] Owens C.A., Peterson C.B., Tang C., Koay E.J., Yu W., Mackin D.S., Li J., Salehpour M.R., Fuentes D.T., Court L.E. (2018). Lung Tumor Segmentation Methods: Impact on the Uncertainty of Radiomics Features for Non-Small Cell Lung Cancer. PLoS ONE.

[7] Yang F., Simpson G., Young L., Ford J., Dogan N., Wang L. (2020). Impact of Contouring Variability on Oncological PET Radiomics Features in the Lung. Sci. Rep..

[8] Baumgartner C.F., Tezcan K.C., Chaitanya K., Hötker A.M., Muehlematter U.J., Schawkat K., Becker A.S., Donati O., Konukoglu E., Shen D., Liu T., Peters T.M., Staib L.H., Essert C., Zhou S., Yap P.-T., Khan A. (2019). PHiSeg: Capturing Uncertainty in Medical Image Segmentation. Medical Image Computing and Computer Assisted Intervention—MICCAI 2019.

[9] Bakas S., Akbari H., Sotiras A., Bilello M., Rozycki M., Kirby J.S., Freymann J.B., Farahani K., Davatzikos C. (2017). Advancing The Cancer Genome Atlas Glioma MRI Collections with Expert Segmentation Labels and Radiomic Features. Sci. Data.

[10] Aerts H.J.W.L., Wee L., Rios Velazquez E., Leijenaar R.T.H., Parmar C., Grossmann P., Carvalho S., Bussink J., Monshouwer R., Haibe-Kains B. Data From NSCLC-Radiomics 2019. https://wiki.cancerimagingarchive.net/display/Public/NSCLC-Radiomics.

[11] Clark K., Vendt B., Smith K., Freymann J., Kirby J., Koppel P., Moore S., Phillips S., Maffitt D., Pringle M. (2013). The Cancer Imaging Archive (TCIA): Maintaining and Operating a Public Information Repository. J. Digit. Imaging.

[12] Armato Samuel G., McLennan G., Bidaut L., McNitt-Gray M.F., Meyer C.R., Reeves A.P., Zhao B., Aberle D.R., Henschke C.I., Hoffman E.A. Data From LIDC-IDRI 2015. https://wiki.cancerimagingarchive.net/display/Public/LIDC-IDRI.

[13] Armato S.G., McLennan G., Bidaut L., McNitt-Gray M.F., Meyer C.R., Reeves A.P., Zhao B., Aberle D.R., Henschke C.I., Hoffman E.A. (2011). The Lung Image Database Consortium (LIDC) and Image Database Resource Initiative (IDRI): A Completed Reference Database of Lung Nodules on CT Scans: The LIDC/IDRI Thoracic CT Database of Lung Nodules. Med. Phys..

[14] Bilic P., Christ P.F., Vorontsov E., Chlebus G., Chen H., Dou Q., Fu C.-W., Han X., Heng P.-A., Hesser J. (2019). The Liver Tumor Segmentation Benchmark (LiTS). arXiv.

[15] Heller N., Sathianathen N., Kalapara A., Walczak E., Moore K., Kaluzniak H., Rosenberg J., Blake P., Rengel Z., Oestreich M. (2020). The KiTS19 Challenge Data: 300 Kidney Tumor Cases with Clinical Context, CT Semantic Segmentations, and Surgical Outcomes. arXiv.

[16] Bakas S., Reyes M., Jakab A., Bauer S., Rempfler M., Crimi A., Shinohara R.T., Berger C., Ha S.M., Rozycki M. (2019). Identifying the Best Machine Learning Algorithms for Brain Tumor Segmentation, Progression Assessment, and Overall Survival Prediction in the BRATS Challenge. arXiv.

[17] Menze B.H., Jakab A., Bauer S., Kalpathy-Cramer J., Farahani K., Kirby J., Burren Y., Porz N., Slotboom J., Wiest R. (2015). The Multimodal Brain Tumor Image Segmentation Benchmark (BRATS). IEEE Trans. Med. Imaging.

[18] Haarburger C., Müller-Franzes G., Weninger L., Kuhl C., Truhn D., Merhof D. (2020). Radiomics Feature Reproducibility under Inter-Rater Variability in Segmentations of CT Images. Sci. Rep..

[19] Virtanen P., Gommers R., Oliphant T.E., Haberland M., Reddy T., Cournapeau D., Burovski E., Peterson P., Weckesser W., Bright J. (2020). SciPy 1.0: Fundamental Algorithms for Scientific Computing in Python. Nat. Methods.

[20] Liljequist D., Elfving B., Skavberg Roaldsen K. (2019). Intraclass Correlation—A Discussion and Demonstration of Basic Features. PLoS ONE.

[21] van Griethuysen J.J.M., Fedorov A., Parmar C., Hosny A., Aucoin N., Narayan V., Beets-Tan R.G.H., Fillion-Robin J.-C., Pieper S., Aerts H.J.W.L. (2017). Computational Radiomics System to Decode the Radiographic Phenotype. Cancer Res..

[22] Traverso A., Wee L., Dekker A., Gillies R. (2018). Repeatability and Reproducibility of Radiomic Features: A Systematic Review. Int. J. Radiat. Oncol. Biol. Phys..

[23] Carrasco J.L., Jover L. (2003). Estimating the Generalized Concordance Correlation Coefficient through Variance Components. Biometrics.

[24] Cox D.R., Kotz S., Johnson N.L. (1992). Regression Models and Life-Tables. Breakthroughs in Statistics.

[25] Pölsterl S. (2020). Scikit-Survival: A Library for Time-to-Event Analysis Built on Top of Scikit-Learn. J. Mach. Learn. Res..

[26] van Velden F.H.P., Kramer G.M., Frings V., Nissen I.A., Mulder E.R., de Langen A.J., Hoekstra O.S., Smit E.F., Boellaard R. (2016). Repeatability of Radiomic Features in Non-Small-Cell Lung Cancer [18F]FDG-PET/CT Studies: Impact of Reconstruction and Delineation. Mol. Imaging Biol..

[27] Suter Y., Knecht U., Alão M., Valenzuela W., Hewer E., Schucht P., Wiest R., Reyes M. (2020). Radiomics for Glioblastoma Survival Analysis in Pre-Operative MRI: Exploring Feature Robustness, Class Boundaries, and Machine Learning Techniques. Cancer Imaging.

[28] Kadoya N., Tanaka S., Kajikawa T., Tanabe S., Abe K., Nakajima Y., Yamamoto T., Takahashi N., Takeda K., Dobashi S. (2020). Homology-based Radiomic Features for Prediction of the Prognosis of Lung Cancer Based on CT-based Radiomics. Med. Phys..

[29] Fu J., Singhrao K., Zhong X., Gao Y., Qi S.X., Yang Y., Ruan D., Lewis J.H. (2021). An Automatic Deep Learning–Based Workflow for Glioblastoma Survival Prediction Using Preoperative Multimodal MR Images: A Feasibility Study. Adv. Radiat. Oncol..

[30] Truhn D., Schrading S., Haarburger C., Schneider H., Merhof D., Kuhl C. (2019). Radiomic versus Convolutional Neural Networks Analysis for Classification of Contrast-Enhancing Lesions at Multiparametric Breast MRI. Radiology.

[31] Huang L., Chen J., Hu W., Xu X., Liu D., Wen J., Lu J., Cao J., Zhang J., Gu Y. (2019). Assessment of a Radiomic Signature Developed in a General NSCLC Cohort for Predicting Overall Survival of ALK-Positive Patients With Different Treatment Types. Clin. Lung Cancer.

[32] Shi Z., Zhovannik I., Traverso A., Dankers F.J.W.M., Deist T.M., Kalendralis P., Monshouwer R., Bussink J., Fijten R., Aerts H.J.W.L. (2019). Distributed Radiomics as a Signature Validation Study Using the Personal Health Train Infrastructure. Sci. Data.

[33] Haarburger C., Weitz P., Rippel O., Merhof D. Image-Based Survival Prediction for Lung Cancer Patients Using CNNS. Proceedings of the 2019 IEEE 16th International Symposium on Biomedical Imaging (ISBI 2019).

[34] Meyer M., Ronald J., Vernuccio F., Nelson R.C., Ramirez-Giraldo J.C., Solomon J., Patel B.N., Samei E., Marin D. (2019). Reproducibility of CT Radiomic Features within the Same Patient: Influence of Radiation Dose and CT Reconstruction Settings. Radiology.

[35] Zhao B., Tan Y., Tsai W.-Y., Qi J., Xie C., Lu L., Schwartz L.H. (2016). Reproducibility of Radiomics for Deciphering Tumor Phenotype with Imaging. Sci. Rep..

[36] Lambin P., Leijenaar R.T.H., Deist T.M., Peerlings J., de Jong E.E.C., van Timmeren J., Sanduleanu S., Larue R.T.H.M., Even A.J.G., Jochems A. (2017). Radiomics: The Bridge between Medical Imaging and Personalized Medicine. Nat. Rev. Clin. Oncol..

[37] Yip S.S.F., Aerts H.J.W.L. (2016). Applications and Limitations of Radiomics. Phys. Med. Biol..

